# CIP2A Promotes T-Cell Activation and Immune Response to *Listeria monocytogenes* Infection

**DOI:** 10.1371/journal.pone.0152996

**Published:** 2016-04-21

**Authors:** Christophe Côme, Anna Cvrljevic, Mohd Moin Khan, Irina Treise, Thure Adler, Juan Antonio Aguilar-Pimentel, Byron Au-Yeung, Eleonora Sittig, Teemu Daniel Laajala, Yiling Chen, Sebastian Oeder, Julia Calzada-Wack, Marion Horsch, Tero Aittokallio, Dirk H. Busch, Markus W. Ollert, Frauke Neff, Johannes Beckers, Valerie Gailus-Durner, Helmut Fuchs, Martin Hrabě de Angelis, Zhi Chen, Riitta Lahesmaa, Jukka Westermarck

**Affiliations:** 1 Turku Centre for Biotechnology, University of Turku and Åbo Akademi University, Turku, Finland; 2 Turku Doctoral Programme of Molecular Medicine (TuDMM), Medical Faculty, University of Turku, Turku, Finland; 3 German Mouse Clinic, Helmholtz Zentrum München, German Research Center for Environmental Health, Neuherberg, Germany; 4 Institute for Medical Microbiology, Immunology and Hygiene, Technische Universität München, Munich, Germany; 5 Department of Dermatology and Allergy, Biederstein, Klinikum rechts der Isar, Technische Universität München (TUM), Munich, Germany; 6 Howard Hughes Medical Institute, Rosalind Russell Medical Research Center for Arthritis, Department of Medicine, Department of Microbiology and Immunology, University of California San Francisco, San Francisco, California, United States of America; 7 Department of Mathematics and Statistics, University of Turku, Turku, Finland; 8 Center of Allergy and Environment Munich (ZAUM), Technische Universität München (TUM), and Institute for Allergy Research, Helmholtz Zentrum München, German Research Center for Environmental Health, Munich/Neuherberg, Germany; 9 Kühne Foundation, Christine Kühne Center for Allergy Research and Education (CK-CARE), Munich, Germany; 10 Institute of Pathology, Helmholtz Zentrum München, German Research Center for Environmental Health, Neuherberg, Germany; 11 Institute of Experimental Genetics, Helmholtz Zentrum München, German Research Center for Environmental Health, Neuherberg, Germany; 12 Institute for Molecular Medicine Finland (FIMM), University of Helsinki, Helsinki, Finland; 13 Clinical Research Group Molecular Allergology, Center of Allergy and Environment Munich (ZAUM), Technische Universität München (TUM), and Institute for Allergy Research, Helmholtz Zentrum München, German Research Center for Environmental Health, Munich/Neuherberg, Germany; 14 Chair of Experimental Genetics, Center for Life and Food Sciences Weihenstephan, Technische Universität München, Freising-Weihenstephan, Germany; 15 German Center for Diabetes Research (DZD), Neuherberg, Germany; 16 Department of Pathology, University of Turku, Turku, Finland; University of Illinois at Chicago College of Medicine, UNITED STATES

## Abstract

The oncoprotein Cancerous Inhibitor of Protein Phosphatase 2A (CIP2A) is overexpressed in most malignancies and is an obvious candidate target protein for future cancer therapies. However, the physiological importance of CIP2A-mediated PP2A inhibition is largely unknown. As PP2A regulates immune responses, we investigated the role of CIP2A in normal immune system development and during immune response *in vivo*. We show that CIP2A-deficient mice (CIP2A^HOZ^) present a normal immune system development and function in unchallenged conditions. However when challenged with *Listeria monocytogenes*, CIP2A^HOZ^ mice display an impaired adaptive immune response that is combined with decreased frequency of both CD4^+^ T-cells and CD8^+^ effector T-cells. Importantly, the cell autonomous effect of CIP2A deficiency for T-cell activation was confirmed. Induction of CIP2A expression during T-cell activation was dependent on Zap70 activity. Thus, we reveal CIP2A as a hitherto unrecognized mediator of T-cell activation during adaptive immune response. These results also reveal CIP2A^HOZ^ as a possible novel mouse model for studying the role of PP2A activity in immune regulation. On the other hand, the results also indicate that CIP2A targeting cancer therapies would not cause serious immunological side-effects.

## Introduction

Despite a considerable improvement in cancer treatment efficiency in the last decade, infectious complications will lead to morbidity and mortality of many patients [1, 2]. These infections, occurring in solid organ tumors and more frequently in hematological malignancies, are often related to treatment-induced immunosuppression [3]. Therefore, the impact of any cancer therapy on patient’s immune system should be evaluated.

Our laboratory previously identified an endogenous PP2A inhibitor protein, CIP2A (Cancerous Inhibitor of PP2A) [4]. To date, numerous studies have demonstrated that CIP2A is a critical inhibitor of PP2A tumor suppressor activity in various human cancer types [5]. CIP2A binds to the PP2A complex, and inhibition of CIP2A in cultured cells increases the catalytic activity of PP2A [4, 6, 7]. Moreover, CIP2A inhibits PP2A-mediated dephosphorylation of several signaling proteins including Akt, DapK and Rictor [6, 8, 9] as well as transcription factors MYC and E2F1 [4, 5, 10]. Importantly, a number of recent studies have demonstrated that the effects of CIP2A depletion on various cellular phenotypes could be rescued via inhibition of PP2A. [10, 11]. However, despite its emerging role as a human oncoprotein and future target for cancer therapies, very little is known about the physiological role of CIP2A. The only physiological function identified thus far by using CIP2A-deficient genetrap mouse model (CIP2A^HOZ^) is CIP2A´s involvement in spermatogenesis [12]. Utilizing a variety of murine models, several independent laboratories have highlighted a potentially important role for PP2A in the regulation of immune responses involved in both autoimmunity and tumor immunity [13–16]. Consequently, we decided to investigate the possible function for CIP2A in the regulation of immune responses. To identify novel physiological functions for CIP2A, CIP2A^HOZ^ mouse model was subjected to a systemic phenotyping screen at the German Mouse Clinic [17, 18], including thorough immunological characterization. Based on that, we report here that CIP2A expression is a novel mechanism important for optimal *in vivo* adaptive immune response after *Listeria monocytogenes (L*.*m*.) re-infection. These data may also have importance in the evaluation of potential immunological effects of future CIP2A-targeted cancer therapies

## Results

### CIP2A is expressed in lymphoid organs but is dispensable for immune system homeostasis

**Fig 1:**
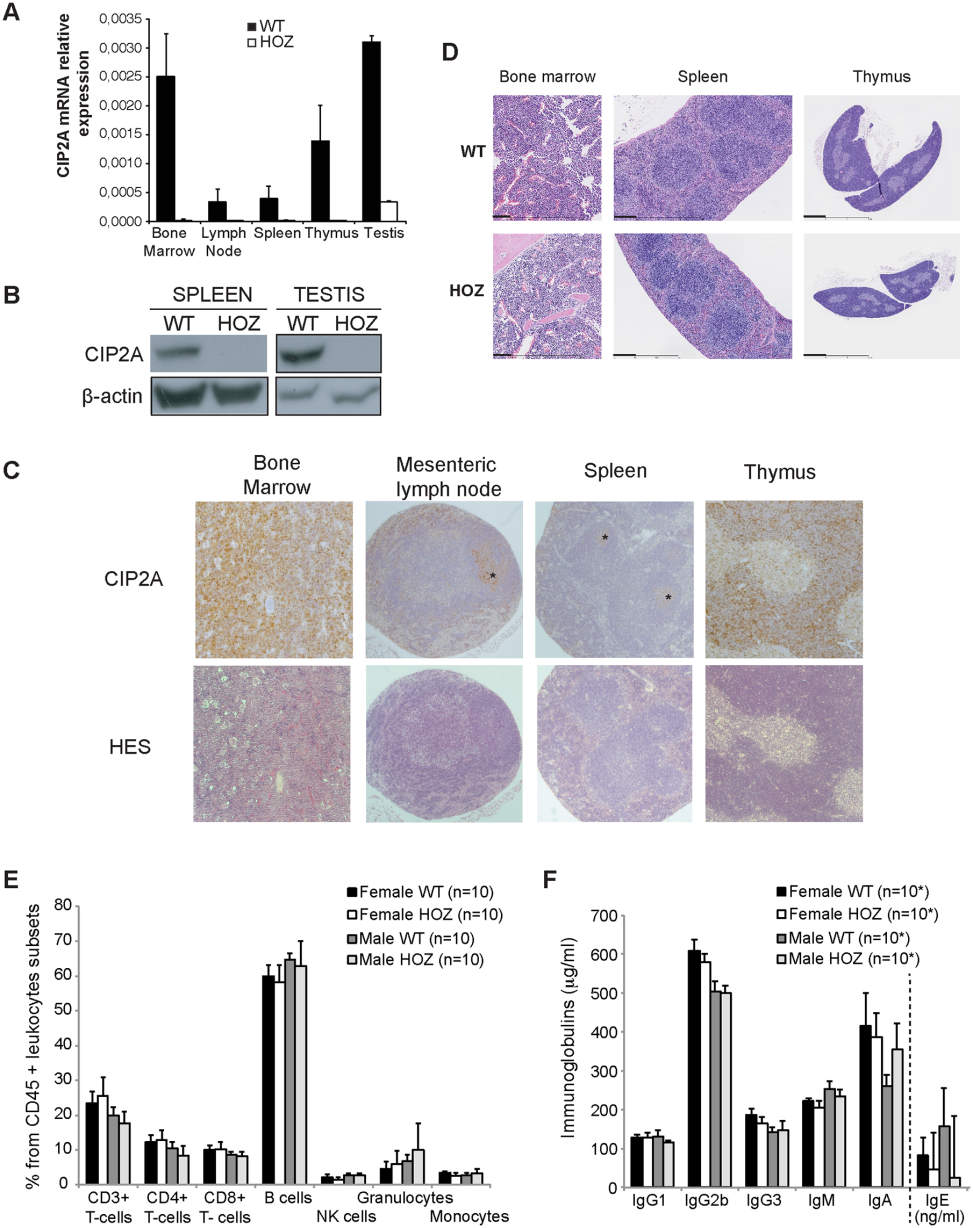
Expression of CIP2A by murine lymphoid organs does not impact the immune system of unchallenged CIP2A^HOZ^ animals. (A) Semi-quantitative RT-PCR for CIP2A RNA in main lymphoid organs and testis from WT and CIP2A^HOZ^ adult mice. Average expression from at least 3 mice (despite testis; S1 Table). (B) Protein expression of spleen and testis from WT and CIP2A^HOZ^ adult mice. Mouse beta-actin was used for normalization. (C) CIP2A-IHC and hematoxilin-eosin staining of common lymphoid organs from adult wild-type mice. Asterix indicate CIP2A expression in germinal centers of lymph nodes and spleen. (D) Hematoxilin and eosin immunohistological staining reveals normal morphology of lymphoid tissues in control and CIP2A^HOZ^ mice. Thick scale bar: 100 μm (Bone marrow); 250 μm (Spleen); 1 mm (Thymus). (E) Flow cytometry analysis of frequencies of leukocyte subpopulations in peripheral blood of WT or CIP2A^HOZ^ females and males (10 mice per condition). (F) Principal immunoglobulins content (μg/ml; except for IgE, ng/ml) in the peripheral blood of WT or CIP2A^HOZ^ adult mice (*: 10 mice per condition, except for IgE, with samples from 9 WT or CIP2A^HOZ^ females, 9 WT males and 7 CIP2A^HOZ^ males). (E, F) Medians are represented and p-values calculated based on a Wilcoxon rank-sum test.

Comparison of CIP2A mRNA expression between different mouse tissues shows the highest level of expression in testis (Fig 1A) [4, 12]. However the lymphoid organs also prominently express CIP2A, both at the mRNA and protein level (Fig 1A and 1B and S1A Fig). As expected, CIP2A expression was not detected in lymphoid tissues from CIP2A^HOZ^ mice (Fig 1A and 1B and S1A Fig) indicating that CIP2A is efficiently deleted in this genetrap mouse model. Immunohistochemical (IHC) staining of WT animals indicate a broad expression of CIP2A in bone marrow and thymus, whereas in spleen and mesenteric lymph nodes, CIP2A was expressed selectively in germinal centers (Fig 1C).

Despite the high expression of CIP2A in primary lymphoid tissues, no pathological alterations were detected in the bone marrow, spleen or thymus from CIP2A deficient mice (Fig 1D). In fact, our histological analysis of 42 different organs revealed no pathological alterations in any of the CIP2A deficient tissues examined (Table 1).

**Table 1:** Histological analysis CIP2A^HOZ^ mice.

Organ	Phenotype	Organ	Phenotype
Adrenal gland	No	Ovaries	No
Blood vessels	No	Pancreas	No
Bone	No	Parathyroid	No
Brain	No	Pituitary gland	No
Cartilages	No	Prostate	No
Cerebellum	No	Rectum	No
Cervix	No	Salivary glands	No
Colon	No	Skeletal muscle	No
Duodenum	No	Skin	No
Epididymis	No	Spinal cord	No
Esophagus	No	Spleen	No
Eyes	No	Stomach	No
Funiculus spermaticus	No	Teeth	No
Heart	No	Testis	No
Jejunum	No	Thymus	No
Kidneys	No	Thyroid	No
Liver	No	Tongue	No
Lung	No	Trachea	No
Lymph nodes	No	Urinary bladder	No
Male accessory glands	No	Uterus	No
Mammary gland	No	Vagina	No

Haematoxilin-eosin stained sections of all organs did not reveal any pathological alterations in the CIP2A deficient mice. 12 CIP2A^HOZ^ mice (6 females, 6 males) and 12 WT mice (6 females, 6 males) were analyzed.

Moreover, CIP2A deficiency in the lymphoid organs did not induce changes in distributions of the main leukocyte populations. In peripheral blood, the frequency of T-cells, B -cells, natural killer cells, granulocytes and monocytes were similar between WT and CIP2A^HOZ^ mice in both sexes (Fig 1E). In a more detailed analysis, only a slight increase in the frequency of CD3^+^gammadeltaTCR^+^ and CD44^+^ T-cells were detected in CIP2A^HOZ^ mice (Table 2), whereas no significant differences between CIP2A^HOZ^ mice and controls were observed in the pattern of B-cell subtypes (S1B Fig). Consistent with unaltered B-cell populations, unchallenged CIP2A^HOZ^ and WT mice had similar serum immunoglobulin (Ig) levels (Fig 1F). We conclude that in spite of a high expression of CIP2A in primary lymphoid tissues, unchallenged CIP2A^HOZ^ mice present normal immune system function.

**Table 2:** Characterization of CIP2A^HOZ^ lymphocytes.

	Male		Female		Linear model		
	HOZ	WT	HOZ	WT	Genotype:Sex	Sex	Genotype
	n = 10	n = 10	n = 10	n = 10			
	mean ± sd	mean ± sd	mean ± sd	mean ± sd	p-value	p-value	p-value
CD45+/T	17.53 ± 3.5	19.83 ± 2.5	25.49 ± 5.5*	23.45 ± 3.5	0,088	**< 0.001**	0,917
CD45+/CD3+CD4+	8.42 ± 2.8	10.39 ± 1.8	12.91 ± 2.7*	12.19 ± 2.1	0,082	**< 0.001**	0,409
CD45+/CD3+CD8+	8.21 ± 1.4	8.7 ± 0.9	10.25 ± 2.1*	9.97 ± 1.3	0,422	**0,001**	0,822
CD45+/B-cells	62.89 ± 7.1	64.73 ± 1.8	58.12 ± 4.9*	59.98 ± 3.2*	0,996	**0,004**	0,237
CD45+/CD5-NK+	2.84 ± 0.3	2.81 ± 0.4	1.52 ± 0.6*	2.12 ± 0.8*	0,088	**< 0.001**	0,112
CD45+/CD11b+Gr1+	10.01 ± 7.7	6.74 ± 1.9	5.92 ± 3.8*	4.57 ± 2.1*	0,523	**0,044**	0,131
CD45+/NK-Gr1-CD11b+	3.34 ± 1.2	2.79 ± 0.6	2.63 ± 0.9*	3.42 ± 0.6*	**0,023**	0,871	0,667
CD3+CD4+/CD25+	5.88 ± 1.3	5.69 ± 0.5	4.37 ± 0.7*	4.41 ± 0.6	0,673	**< 0.001**	0,782
CD45+/CD3+ γδ TCR+	0.32 ± 0.1	0.24 ± 0	0.47 ± 0.1*	0.42 ± 0.1	0,629	**< 0.001**	**0,018**
CD3+CD4+/CD62L+	60.24 ± 17	63.51 ± 15.6	65.92 ± 10.6*	58.86 ± 15.7	0,291	0,915	0,696
CD3+CD4+/CD44+	69.76 ± 7.3	65.03 ± 3.7	72.54 ± 2.9*	67.82 ± 1.9	0,998	0,061	**0,002**
CD3+CD8+/CD62L+	75.8 ± 15.3	76.88 ± 14	79.11 ± 10.7*	73.84 ± 13.9	0,474	0,976	0,636
CD3+CD8+/CD44+	67.94 ± 9.6	64.38 ± 2.9	66.7 ± 2.7*	63.4 ± 3.4	0,942	0,535	0,061
CD45+/CD5+NK+	0.09 ± 0	0.11 ± 0	0.12 ± 0.1*	0.11 ± 0*	0,422	0,343	0,608
B-cells/IgD+	90.57 ± 3.5	92.12 ± 0.9	83.96 ± 5.7*	85.47 ± 2.3*	0,986	**< 0.001**	0,187
B-cells/CD5+	1.32 ± 0.5	1.55 ± 0.4	1.71 ± 0.3*	1.58 ± 0.2*	0,163	0,112	0,686
B-cells/B220+MHCclassII+	63.6 ± 7.7	61.54 ± 5.3	29.99 ± 17.6*	30.59 ± 13.2*	0,729	**< 0.001**	0,849
NK+/CD11b+	83.84 ± 5.5	81.15 ± 3.5	86.58 ± 3.2*	79.32 ± 28	0,63	0,924	0,297
CD4/CD8	1.05 ± 0.33	1.19 ± 0.13	1.27 ± 0.11*	1.22 ± 0.07	0,122	0,05	0,437

Leukocyte subset frequency (% of CD45^+^ cells or respective subset) mean, standard deviation and p-value calculated by a linear model (parameter ~ genotype x sex);

* number based on 10 WT or CIP2A^HOZ^ males and 9 WT or CIP2A^HOZ^ females.

### CIP2A is required for optimal systemic immune response

**Fig 2:**
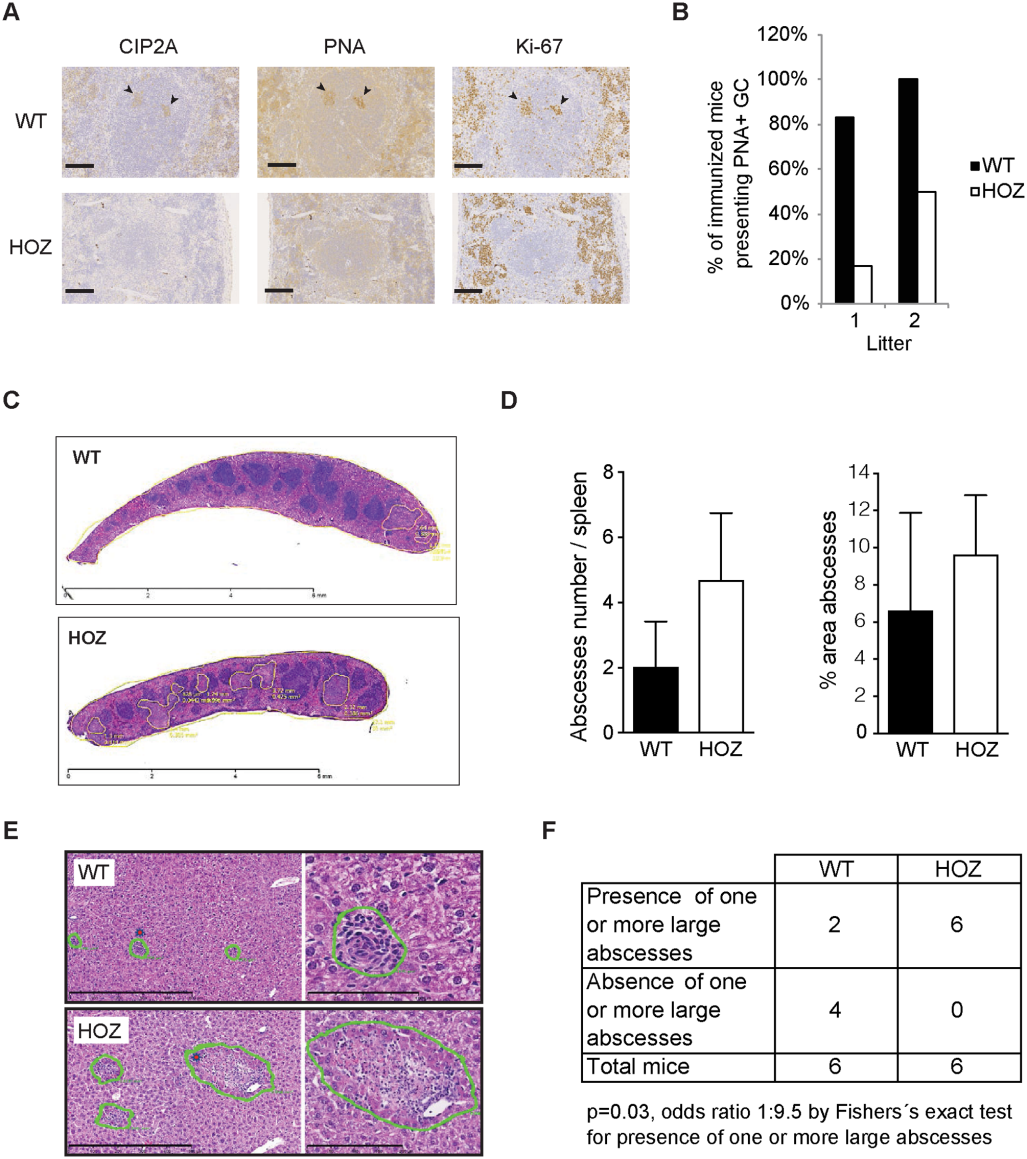
Impaired adaptive immune response in CIP2A deficient mice. (A) Immunohistochemical staining for CIP2A, germinal center marker peanut agglutinin (PNA), and proliferation marker Ki-67 in spleen from immunized WT and CIP2A^HOZ^ mice. Arrows indicate positive staining of germinal center of indicated antigens in WT tissues. Scale bar represents 100 μm. (B) Percentage of immunized mice presenting a PNA positive staining in germinal center from two independent mouse cohorts of 6 WT and 6 CIP2A^HOZ^ females, and 7 WT and 6 CIP2A^HOZ^ females respectively. (C) Haematoxilin histological staining of spleen after primary infection with *L*.*m*. Bacterial abscesses are highlighted in yellow. Scale bar: 6 mm. (D) Abscesses number and percentage of the abscesses area compared to spleen total area after *L*.*m*. primary infection. Average of 2 WT and 3 CIP2A^HOZ^ females is shown. Mann-Whitney t test. (E) Hematoxilin-eosin histological staining of liver presenting inflammatory lesions developed in response to second infection with intracellular bacteria *L*.*m* (5 days post recall infection). The right panels present higher amplifications of the lesions on the left. Scale bar represents 500 μm (left panel) or 100 μm (right panel). Representative pictures of 6 WT and 6 CIP2A^HOZ^ females analyzed. (F) Quantitative and statistical analysis of the number of large abscesses (> 10 μm^2^) as described in (E).

However, the analysis of the gene expression profile of the spleen from CIP2A^HOZ^ mice revealed differential expression of genes related to the regulation of immune response and/or autoimmune diseases (Table 3). Motivated by these findings, we performed functional *in vivo* assays to elucidate the role of CIP2A within an immune response. To start with, mice were immunized with ovalbumin in order to measure the T-cell-dependent antibody response. Compared to WT mice, the induction of peanut agglutinin (PNA) positivity and proliferation in germinal centers was attenuated in CIP2A^HOZ^ mice (Fig 2A and 2B) indicating a role of CIP2A within the T-cell dependent B-cell response.

**Table 3:** Molecular function of significantly regulated genes identified via GO term enrichment analysis of CIP2A^HOZ^ versus WT spleens. CIP2A is the only gene downregulated in CIP2A^HOZ^ samples, all the other genes were found to be up-regulated in mutant samples when compared to WT.

Category	Regulated genes
tumorigenesis	Actc1, Ccr5, CD22, Clec7a, Csrp3, Cxadr, Dek, Eif5, Elmo1, Fam65b, Lpcat2, Lypla1, Myl2, Nrn1, Spink1, Stap1, Tank, Tff2, Tnfrsf13b, Tram1, Ucp1, Vegfa, Zg16
**immune response**	**Ccr5, CD22, CD96, Clec7a, Cxadr, Dek, Elmo1, Gpx4, Nppa, Tank,Tff2, Tnfrsf13b, Vegfa**
metabolism of protein	CD22, Ctrb2, Ctrl, Dars, Eif5, Klk1b1, Nppa, Spink1, Sumo1, Sycn, Vegfa
morphology of organ	Actc1, Clec7a, Csrp3, Cxadr, Elmo1, Klk1b1, Nppa, Spink1, Tff2, Tnfrsf13b, Vegfa
**autoimmune disease**	**Adss, Ccr5, CD22, Clec4a, Dek, Tank, Tnfrsf193, Tram1, Usp15, Vegfa**
cellular homeostasis	Ccr5, CD22, Clec7a, Csrp3, Klk1b1, Myl3, Nppa, Tnfrsf13b, Ucp1, Vegfa
cell surface receptor linked signal transduction	Ccr5, CD22, Clec4a, Nppa, Nsdhl, Stap1, Tnfrsf13b
organization of cytoskeleton	Ccr5, Csnk1d, Cxadr, Elmo1, Myl2, Snx2, Vegfa

As innate and adaptive immune responses are required for the clearance of intracellular bacteria *L*.*m*. [19] [20], WT and CIP2A^HOZ^ mice were infected with *L*.*m* (S2A Fig). To investigate the impact of CIP2A in response to primary infection, appearance of spleen and liver abscesses as a sign of un-cleared infection, were examined 3 days post-infection (S2A Fig). Even though CIP2A^HOZ^ mice presented with more splenic bacterial abscesses and larger infected areas as compared to WT mice, this difference was not statistically significant (Fig 2C and 2D and S2B Fig). These large necrotic lesions (abscesses) consist of granulocytes and/or exudate macrophages (S2B Fig). Small lesions containing mainly mature macrophages (microgranulomas) were detected in lower proportion as well (data not shown).

To assess the role of CIP2A within the course of an adaptive immune response, mice were injected with a sub-lethal infection dose of *L*.*m*.-OVA, followed 4 weeks later by a challenge with a lethal dose of the same bacteria (S2A Fig). Primary infection induced a protective memory response leading to bacteria clearance as no abscesses were detectable histologically in spleen and a decrease in the number of necrotic lesions and an increase in the number of small granulomatous lesions in the liver of infected mice in both genotypes was observed. Notably, after the second infection with a lethal dose, when compared to WT mice, CIP2A^HOZ^ mice did present a significant increase in large bacterial abscesses in the liver (Fig 2E and 2F) (p = 0.03), as well as a trend for higher splenic bacterial load (S2C Fig), indicating for a reduced adaptive immune response in CIP2A-deficient mice.

We conclude that, despite CIP2A being dispensable for normal development and function of the immune system in unchallenged conditions, CIP2A does contribute to a certain extent to the T-cell dependent immune response *in vivo*.

### Impaired *in vivo* T-cell activation in CIP2A-deficient mice

**Fig 3:**
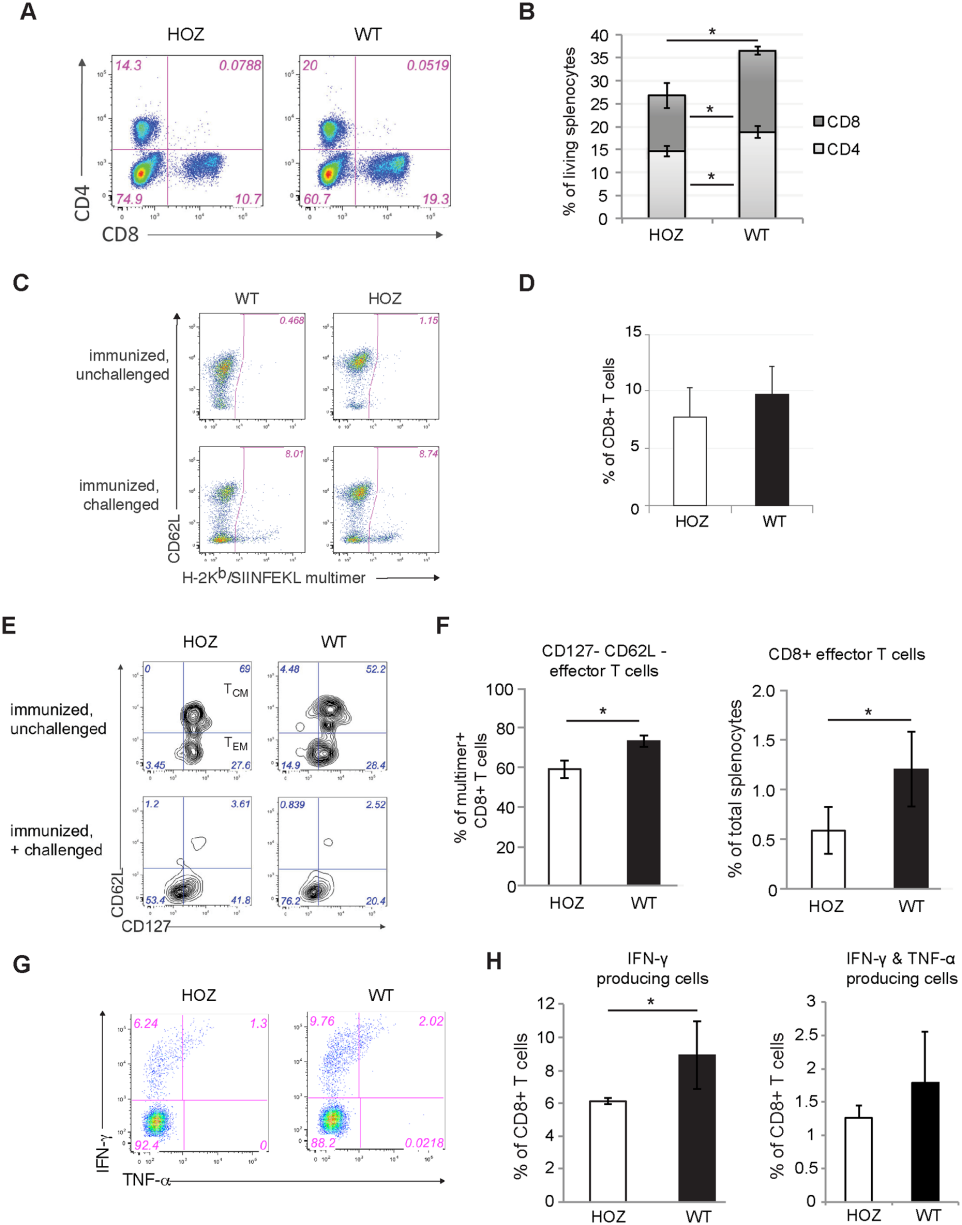
Impaired T-cell activation in CIP2A^HOZ^ mice *in vivo*. (A-H) Flow cytometry analysis of splenocytes from 3 WT and 4 CIP2A^HOZ^ mice five days after recall infection with high-dose *L*.*m*.-OVA. (A) Representative flow cytometry analysis for CD4^+^ and CD8^+^ T cells. (B) Percentage of CD4^+^ and CD8^+^ splenocytes from analysis described in (A). * p < 0.05, Two-tailed T-test. (C) Representative dot plots of antigen-specific CD8^+^ T cells identified by H-2K^b^/SIINFEKL multimer staining. Dot plots are gated on living CD45^+^ CD3^+^ CD8^+^ cells. (D) Bar chart numbers indicate H2-Kb/SIINFEKL multimer+ cells as percentages of CD8+ T cells. T-test. (E-H) Analysis of OVA-specific CD8^+^ T lymphocytes from control and mutant mice 5 days after recall infection with high-dose *L*.*m*.-OVA and unchallenged control mice. (E) CD62L and CD127 surface expression on OVA-specific (H-2K^b^/SIINFEKL multimer^+^) allows the characterization of secondary T-cell subsets: central memory phenotype (T_CM_, CD127^+^ CD62L^+^) and effector memory phenotype (T_EM_, CD127^+^ CD62L^-^) can be observed in immunized, unchallenged mice; whereas immunized challenged mice present two main populations of effector T cells differentiated by their CD127 expression. (F) Bar chart numbers indicate percentages of effector phenotype CD8^+^ T-cells in regards of total splenocytes or OVA-specific CD8^+^ T lymphocytes (right). * p < 0.05, Two-tailed T-test. (G) Cytokine production by antigen-specific T-cells from CIP2A^HOZ^ and WT mice on day 5 after recall infection with high-dose *L*.*m*.-OVA. (H) Proportion of T_EM_ and T_CM_ from total splenocytes. * p < 0.05, Two-tailed T-test.

During *L*.*m*. infection, both CD4^+^ and CD8^+^ T-cells are activated and these cells comprise most of the adaptive immune response [19]. Notably, on day 5 after *L*.*m*. reinfection, a significant reduction in number of both CD4^+^ and CD8^+^ T cells was detected among CIP2A^HOZ^ splenocytes (Fig 3A and 3B and S3A Fig). Moreover, immunized challenged CIP2A^HOZ^ mice presented a reduced number of *L*.*m*-specific CD8^+^ T cells when compared to WT counterparts (S3B Fig). However, we did not observe a statistically significant decrease in the percentage of *L*.*m*-specific CD8^+^ T cells in immunized challenged CIP2A deficient mice (Fig 3C and 3D). Nevertheless, the characterization of T cell subsets revealed a decreased frequency of the CD8^+^ T cells with a secondary effector phenotype (CD127^-^ CD62L^-^) in immunized challenged CIP2A deficient mice (Fig 3E and 3F). Concomitantly, we observed a reduction of the frequency of IFN-γ producing CD8^+^ T cells in these immunized challenged CIP2A^HOZ^ mice (Fig 3G and 3H), whereas TNF-α secretion of CD8+ T-cells or IFN-γ and TNF-α co-production of CD4+ T-cells were not affected by CIP2A deficiency (S3C and S3D Fig). Notably, CD8^+^ T cell populations with secondary effector memory phenoptype (CD127^+^CD62L^-^) and central memory phenotype (CD127^+^CD62L^+^) were not reduced in immunized challenged CIP2A^HOZ^ mice (S3E Fig). We conclude that CIP2A influences T cell responses *in vivo*, possibly by promoting the generation of CD8^+^ effector T-cells and the generation of IFN-γ producing CD8+ T-cells.

### Cell autonomous function for CIP2A in T-cell activation

**Fig 4:**
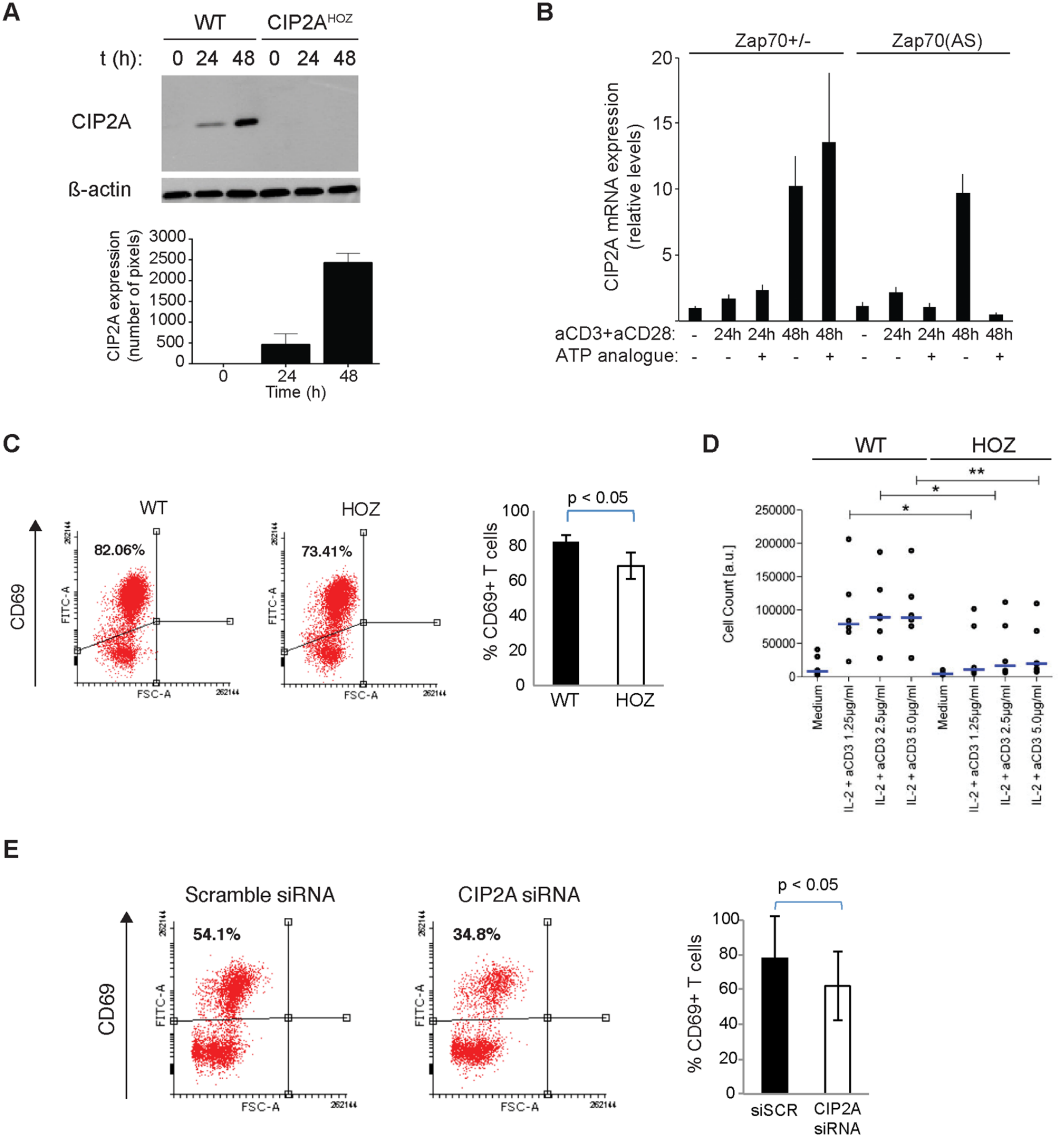
Cell autonomous function for CIP2A in T-cell activation. (A) CIP2A protein expression from activated WT or CIP2A^HOZ^ CD8 T-cells. Mean + S.E.M of CIP2A protein expression using β-actin as a normalization control is shown. (B) CD4^+^ cells from Zap70^+/-^ or ATP analogue HXJ2-sensitive Zap70^(AS)^ mice were stimulated *in vitro* with plate-bound anti-CD3 and anti-CD28 antibodies in the presence or absence of HXJ42 (1 μM). Cells were harvested at indicated time-points and shown is real-time PCR analysis of CIP2A transcript levels relative to actin as normalized to the Zap70^+/-^ unstimulated sample. Shown is a representative of two independent experiments with identical results. (C) Cell surface staining of CD69 from CD4^+^CD62L^+^ T-cells isolated from WT or CIP2A^HOZ^ mice stimulated with anti-CD3 and anti-CD28 for 24h. The mean + S.E.M. of three independent experiments is shown. Student's t test. (D) Number of viable splenocytes determined by CellTiter-Glo Assay 7 days post-stimulation with IL-2 (20U/ml) and anti-CD3 (1.25, 2.5 or 5 μg/ml). Blue bars indicate medians, circle individual data points (n = 6 for WT & CIP2A^HOZ^ cells). * p<0.05, ** p<0.01, Student’s t-test. (E) Human CD4^+^ T-cells isolated from umbilical cord blood pooled from 5–6 individuals were nucleofected with scramble nontargeting siRNA or CIP2A siRNA. Cells were rested for 48hrs and activated with anti-CD3 and anti-CD28 for 24h. The mean + S.E.M. of three independent experiments is shown. Student's t test.

Due to complex nature of interplay between immune cells during the immune response *in vivo*, it is essential to study whether CIP2A regulates T-cell activation in a cell autonomous fashion. Therefore, we first assessed whether CIP2A expression is induced in activated T-cells *in vitro*. Indeed, CIP2A protein expression was induced in WT CD8^+^ T-cells, which was not observed in T-cells from CIP2A deficient mice (Fig 4A). To further investigate the importance of CIP2A on T-cell antigen receptor (TCR) mediated T-cell activation, we isolated murine CD4^+^ T-cells from mice in which one allele of TCR signal transducer Zap70 is deleted (Zap70^+/-^), but which display normal immune response, and compared these to cells from Zap70(AS) mice in which ATP binding pocket of Zap70 is engineered to be selectively inhibited by ATP analogue HXJ [21, 22]. This allows selective inhibition of Zap70 activity during T-cell activation upon HXJ treatment and thus allows the assessment of whether TCR-mediated T-cell activation is linked with CIP2A gene regulation. Treatment with combination of anti-CD3 and anti-CD28 induced CIP2A mRNA expression at 48 hours in both Zap70^+/-^ and control Zap70^(AS)^ CD4 T-cells (Fig 4B). Importantly, CIP2A mRNA induction was completely prevented by HXJ treatment of Zap70^(AS)^ cells, whereas the compound did not affect CIP2A expression in Zap70^+/-^ cells (Fig 4B). These results demonstrate that CIP2A expression is intimately linked with T-cell activation in a cell autonomous fashion. Role of Zap70 in CIP2A regulation is further confirmed by significantly decreased CIP2A mRNA expression in Zap70 negative JurkaT-cells (S4A Fig).

To assess the functional relevance of CIP2A induction for cell autonomous T-cell activation, T-cells isolated from WT or CIP2A^HOZ^ mice were treated with anti-CD3 and anti-CD28 and activation was assessed by ratio of CD69 positive and negative cells. Notably, loss of CIP2A resulted in significant inhibition of T-cell activation (Fig 4C), possibly by reducing proliferation of activated T cells as suggested by CFSE staining (S4B and S4C Fig). To assess the long-term effects of CIP2A loss on T-cell activation, we compared the number of viable cells after *in vitro* activation of WT and CIP2A^HOZ^ splenocytes by IL-2 and anti-CD3. As shown in Fig 4D, number of CIP2A^HOZ^ splenocytes was significantly impaired 7 days post-stimulation.

These results demonstrate that CIP2A promotes T-cell activation in a cell autonomous fashion. Importantly, these results can be also extended to human T-cells as siRNA-mediated inhibition of CIP2A expression significantly inhibited their activation (Fig 4E & S4D and S4E Fig).

## Discussion

Since its original characterization in 2007 as an oncogenic PP2A inhibitor protein [4], CIP2A has been documented to be a clinically relevant oncoprotein in the vast majority of solid and hematological human cancers tested [5]. However, our understanding of CIP2A’s function thus far has been limited to its reported role in promoting mouse spermatogenesis [12]. In the present work, we have thoroughly characterized both CIP2A expression and function in lymphoid tissues and immune cells. CIP2A is expressed in all lymphoid tissues analyzed with highest expression in the bone marrow and thymus. Based on the normal repertoire of all immune cells analyzed, CIP2A expression appears to be dispensable for immune system homeostasis or lymphocyte differentiation. Thus, developmental defects can be excluded as the main cause for impaired lymphocyte activation in CIP2A^HOZ^ mice. Using *L*.*m*. infection as an *in vivo* model to study the impact of CIP2A in immune responses, our results would suggest that CIP2A is dispensable for innate immunity and yet play an important role in the adaptive immune response induced by *L*.*m*. Detailed analysis of T-cell repertoire affected by CIP2A loss during the adaptive immune response against *L*.*m*. revealed that CIP2A supports both CD4^+^ and CD8^+^ T-cell survival. Among the CD8^+^ T-cells, CIP2A was selectively required for the induction of effector T-cells. During infection, a large proportion of *L*.*m*. is phagocyted by resident macrophages in the liver and subsequently, formation of large necrotic abscesses occurs. The infection is controlled by the formation of microgranulomas composed of *L*.*m*.-specific T lymphocytes and activated macrophages secreting cytokines such as IFN-γ [23]. Therefore, the reduced secretion of IFN-γ by *L*.*m*.-specific CD8^+^ T-cells (Fig 3G and 3H) can be phenotypically correlated to the significant increase of large abscesses in the liver of re-challenged CIP2A^HOZ^ mice when compared to WT mice (Fig 2E and 2F).

Together these results firmly establish CIP2A as a novel signaling protein important for T-cell activation *in vivo*. Also, our results demonstrate that CIP2A is downstream of Zap70 activity during T-cell activation (Fig 4B and S4A Fig), which is interesting considering the role for Zap70 in human immune diseases [24]. Moreover, this decreased activation could be due to a reduced proliferation of CIP2A deficient T cells as suggested by CFSE dilution staining (S4D and S4E Fig).

To date, numerous studies have demonstrated that CIP2A is a critical inhibitor of PP2A tumor suppressor activity in various human cancer types [5]. Importantly, CIP2A inhibition increases the catalytic activity of PP2A [4, 6, 7], and several phenotypes induced by CIP2A inhibition can be reversed by co-inhibition of PP2A [10, 11]. Therefore, even though it is impossible to completely rule out potential PP2A-independent mechanisms in mediating the immune response defects in CIP2A^HOZ^ mice, it is very likely that they are linked to PP2A regulation. This conclusion is further supported by previous data that PP2A limits CD28-elicited signal transduction during IL-2-induced T-cell activation [25], which are in perfect concert with observed decrease in IL-2-induced activation in CIP2A deficient T-cells (Fig 4D). On the other hand, our results are in good agreement with recent data that PP2A is inhibited in human autoimmune diseases [15, 26]. Mechanistically, CIP2A-mediated PP2A inhibition could support T-cell activation via its effects on MYC, which is a well-characterized CIP2A-regulated PP2A target [4, 5, 11] and important for T-cell activation [27, 28]. Other CIP2A-regulated effectors previously implicated in T-cell activation include CREB [29], Akt/PKB [6] and mTOR [8, 30].

In summary, our analysis of the potential physiological role for the recently discovered human oncoprotein CIP2A, identifies CIP2A as a novel factor involved in activation of both CD4^+^ and CD8^+^ T-cells and in adaptive immune regulation. In the light of these results, it is well reasoned to further explore the functional relevance of mechanisms regulating PP2A activity for host response to microbial infections. Use of the CIP2A mouse model presented here will provide a feasible tool to advance this understanding. On the other hand, these results give important guidelines to estimate potential immunological side-effects of future CIP2A targeted cancer therapies, which probably would not be as limiting with comparison to reported effects of many existing cancer treatments [1, 2].

## Material and Methods

### Mouse strain

The CIP2A hypomorphic mouse model was previously described [10, 12]. All animal studies were conducted in accordance with the guidelines of the Provincial Government of Southern Finland and handled in accordance with the institutional animal care policies of the University of Turku (license ESLH-2007- 08517).

At GMC, mice were maintained according to the GMC housing conditions and German laws. All tests performed at the GMC were approved by the responsible authority of the Regierung von Oberbayern.

Zap70^+/-^ and Zap70^(AS)^ mice were housed in the specific pathogen-free facility at the University of California, San Francisco, and were treated according to protocols approved by the Institutional Animal Care and Use Committee in accordance with NIH guidelines. Zap70^+/-^ and Zap70^(AS)^ mice were described previously [21, 22].

### Tissues sample homogenization and RNA extraction

Liquid nitrogen frozen murine samples were homogenized using the MagNA Lyser and MagNA Lyser Green Beads (Roche Diagnostics). Briefly, RNA and protein samples were homogeneized in respective lysing buffers, RA1 from Macherey Nagel for RNA; RIPA buffer (PBS; 1% Nonidet P-40; 0.5% sodium deoxycholate; 0.1% SDS) for protein samples. 1 to 4 cycles (6500 rpm, 50 sec) were used to homogenize the tissues, an ice-cooling step of 2 min being done between each cycle. Total RNA was extracted and cleaned up from the lysate using the Nucleospin kit (Macherey Nagel), including a DNAse treatment step.

### Antibodies

Following antibodies were used for Western blotting: rabbit polyclonal anti-CIP2A [31], mouse monoclonal anti-ß-actin, clone AC-74 (Sigma).

### RT-PCR analysis

For cDNA synthesis, 1 μg total RNA was incubated with 250 ng random hexamer for 5 min at 70°C, then cooled down on ice for another 5 min. Total RNA was reverse transcribed in a final volume of 25 μL containing enzyme buffer, 10 units of RNAse inhibitor, DTT, 0,5 mM deoxynucleotide triphosphate, and 200 units MMLV reverse transcriptase (M5301, Promega). The samples were incubated at room temperature for 10 min, then at 42°C for 50 min. The reverse transcriptase was finally inactivated by heating at 70°C for 15 min

PCR amplification: The quantification was based on the standard curve method. The data were normalized using ß-actin. Oligonucleotides were obtained from Proligo. For quantitative real-time PCR, 2 μL of diluted reverse transcription reaction samples (1/10) were added to 8 μL of a PCR mixture made up of 5 μL of PCR Master Mix (Applied Biosystems), 1 μL of each primer at a concentration of 3 μM, and 1 μL of specific probe at a concentration of 2 μM. The thermal cycling conditions comprised an initial step at 50°C for 2 min and a denaturation step at 95°C for 10 min followed by 40 cycles at 95°C for 15 seconds and 60°C for 1 min. All PCRs were carried out using an ABI Prism 7000 Sequence Detection System (Applied Biosystems). The specificity of each primer couple was shown by a dissociation curve analysis. Results are derived from the average of at least two independent experiments. Gene expression was reported relative to housekeeping gene ß -actin. Following primers were used: CIP2A Fwd: 5’- GAACAGATAAGGAAAGAGTTGAGCA -3’, CIP2A Rev: 5’- ACCTTCTAATTGAGCCTTGTGC -3’; ß-actin Fwd: 5’- TGGCTCCTAGCACCATGAAGA -3’, ß-actin Rev: 5’- GTGGACAGTGAGGCCAGGAT -3’.

For real-time PCR analysis from ZAP70 mice, RNA was harvested from unstimulated or stimulated CD4+ cells using the RNeasy kit (Qiagen), and reverse transcribed using the SuperScript III first strand reverse transcriptase kit (Invitrogen). Real-time PCR was performed using the QuantStudio 12K analyzer (Applied Biosystems). Relative quantity (ΔΔCT) values, normalized to actin and Zap70+/- unstimulated cells are shown.

### Immunohistochemistry and tissue samples

Formalin-fixed, paraffin embedded sections of mouse organs were stained with primary antibody CIP2A [31], ki-67 (M7249, Dako) or peanut agglutinin (PNA, L6135, Sigma-Aldrich). Secondary antibody (Dako EnVision anti-rat) or HRP conjugated streptavidin from the LSAB+ skit (K0690, Dako) were used for visualisation. DAB+ liquid Dako (K3468) was used as a substrate for peroxidase. Samples were incubated in Mayers HTX, rinsed and finally dehydrated, cleared and mounted.

### Immunization

A stock solution of chicken egg ovalbumin (Sigma), at 1mg/ml in PBS, was prepared. Then 100 μl of this solution was mixed with 100 μl of Complete Freunds Adjuvant (CFA) per mouse. These 200 μl were intraperitoneally injected using a 30 gauge needle, after sterilizing with alcohol pads the site of injection.

### Immunoglobulins

The plasma levels of IgM, IgG1, IgG2b, IgG3, and IgA were determined simultaneously in the same sample using a bead-based assay [32] with monoclonal anti-mouse antibodies conjugated to beads of different regions (Biorad, USA), and acquired on a Bioplex reader (Biorad). IgE was measured by ELISA. In brief, microtiter plates (96-well) were coated with 10 μg/ml anti-mouse IgE rat monoclonal IgG (clone-PC284, The Binding Site) to detect total IgE.

### Listeria monocytogenes infection

Infection experiments were performed with recombinant *Listeria monocytogenes* expressing ovalbumin (L.m.-Ova, [33]) or the parental wild type *Listeria monocytogenes* strain 10403s (L.m.-wt). Brain Heart Infusion (BHI) medium was inoculated with listeria stock solution and incubated at 37°C until an OD600 of 0.05–0.1. After dilution with PBS to an appropriate concentration the infection of mice was performed with the indicated dose by intravenous (i.v.) injection into the lateral tail vein.

### Pathology and Histological analysis of *Listeria monocytogenes* infected spleen or liver

A total of 40 mice were analysed at the age of 17–20 weeks. The mice were sacrificed with CO2 and visceral organs weighted. 42 Organs were fixed in 4% neutral buffered formalin and embedded in paraffin. Two-μm-thick sections were cut and stained with haematoxylin and eosin (H&E) for histological examination. In a select group of mice challenged with L m. infection (12 CIP2A^HOZ^ and 12 WT female mice) the analysis was complemented by quantification of the area of inflammatory lesions (abscesses) in spleen and liver. Images of tissue sections were acquired with an automated slide scanner (NanoZoomer^®^-HT Hamamatsu, Japan). All slides were independently reviewed and interpreted by two pathologists experienced in mouse pathology. Statistical analysis was performed in an area of 5mm^2^ using the Fisher’s exact test. A statistical significance was considered at p-value (<0,05).

### Isolation and activation of CD8^+^ T-cells

Total CD8^+^ T-cells were isolated from spleen and lymph nodes and cultured as previously described [34]. Briefly, CD8^+^ T-cells were cultured in Dulbecco’s modified Eagle’s medium (DMEM) supplemented with 10% heat-inactivated fetal calf serum, 2 mM L-glutamine, penicillin- streptomycin, non-essential amino acids, sodium pyruvate, vitamins, 10 mM HEPES, and 50 mM 2-mercaptoethanol and stimulated with anti-CD3 (clone 2C11) and anti-CD28 (clone 37.51) (1 mg/ml) for 0h, 24h and 48h.

### Isolation and activation of murine CD4^+^ T-cells

Mouse naive T-cells were isolated from spleens and lymph nodes of gender and age matched wild type or CIP2A^HOZ^ mice by using CD4^+^CD62L^+^ T-cell isolation kit (Miltenyi Biotech). Cells were activated with plate-bound anti-CD3 (1μg/ml) and anti-CD28 (2 μg/ml, both from BD Pharmingen) and cultured in IMDM medium (Gibco, Life Technologies) supplemented with penicillin (100 U/ml)/streptomycin (100 μg/ml) and 10% FCS (Biowest, Nuaillé, France). At 24 hr, cells were collected and stained with anti-mouse CD69-FITC or isotype control antibodies (both are from eBioscience) followed by flow cytometry analysis by FACS Calibur (BD Biosciences). CFSE dilution experiment was performed as previously described [35].

### Isolation and activation of human CD4^+^ T cell

Human CD4^+^T Cells were isolated from the umbilical cord blood collected from healthy neonates in Turku University Hospital, Turku Finland with approval from the Finnish Ethics Committee. Participants provided their verbal informed consent to participate in this study. This was considered more feasible and practical than a written consent. However, midwifes kept records of this consent. Moreover, this consent procedure has been approved by the Finnish Ethics Committee.

Briefly, mononuclear cells were isolated with Ficoll-Paque gradient centrifugation (GE Healthcare Biosciences AB, Uppsala, Sweden). CD4^+^ T cells were separated by using magnetic beads (Dynal CD4 Positive Isolation Kit, Invitrogen, Oslo, Norway).

All human CD4^+^T cells used in this study were pooled samples from 5–6 individuals. Each pool of cord blood CD4+ T cells samples were divided into two aliquots. Down-regulation of CIP2A expression in human CD4^+^ T cells were carried out by introducing CIP2A specific siRNAs in one aliquot (siRNA-1, 5’-CUGUGGUUGUGUUUGCACU-3; siRNA-2, 5’-GCACGGACACUUGCUAGUA-3; siRNA-3, 5’-GUACCACUCUUAUAGAACA-3’) and control non-targeting scramble siRNA into another aliquot of CD4^+^ T cells (4.5 μg siRNA/4x10^6^ cells) using Nucleofector (Amaxa Biosystems, Lonza), after which cells were rested for 48 hr before culturing. Cells were activated with plate-bound anti-CD3 (500 ng/24well culture plate, Immunotech, Marseille, France) and soluble anti-CD28 (1 μg/ml, Immunotech) in Yssel's medium [36] supplemented with 1% human AB serum (Red Cross Finland Blood Service). At 24 hr, cells were collected and CD69-FITC or isotype control (both from BD Biosciences) staining were performed. Expression of CD69 was detected by flow cytometer (FACS LSRII or FACSCalibur both from BD Biosciences) and analyzed by using flowing software. CFSE dilution experiment was performed as previously described [35]. For Western blot detections, cells were collected after 48 hr of activation with anti-CD3/anti-CD28 and were probed with the following antibodies: CIP2A [31], ETS1 (Santa Cruz Biotechnology), MYC (Covance, New Jersey), IRF4 (Santa Cruz Biotechnology), ERK1,2 (Cell Signaling Technology), and ß-actin (Sigma).

### T cell isolation and stimulation (ZAP70 model)

Murine CD4^+^ cells were isolated by negative selection using an antibody cocktail containing biotinylated antibodies against CD8, CD11b, CD11c, CD19, CD24, CD45R, (Tonbo biosciences), DX5 and Ter119 (Biolegend), as well as anti-biotin conjugated magnetic beads (Miltenyi). Isolated CD4^+^ cells were stimulated on tissue culture plates coated with stimulatory antibodies against CD3e (clone 145-2C11) and CD28 (clone 37.51) (both from the UCSF monoclonal antibody core facility). T cells were stimulated in culture for up to 48 hours in media supplemented with analogue inhibitor HXJ42 (1 μM) [37] or vehicle alone (DMSO).

### Cell viability of *in vitro* stimulated murine splenocytes

Spleen tissue from six male *Cip2a*-mutant and six control mice was used in this study. Splenocytes were isolated and cultivated in 96-well plates with 100,000 cells per well. Total splenocytes were incubated with 20 U/ml Il-2 and 5, 2.5, and 1.25 μg/ml antiCD3. After 7 days of cultivation, the number of viable cells was determined by CellTiter-Glo Assay (Promega).

### Transcriptome analysis

Total RNA of spleen from of 17 weeks old male animals (CIPA^HOZ^ n = 4, wild type n = 4) was extracted according a standardized protocol (RNAeasy mini kit, Qiagen). For gene expression profiling, Illumina Mouse Ref8 v2.0 Expression BeadChips were performed as previously described [38]. Illumina Genome studio 2011.1 was applied for data normalization (cubic spline) and background corrections. Statistical analysis identified differential gene expression between mutant and wild type tissue comparing single mutant values with the mean of four wild types (FDR < 10% in combination with mean fold change > 1.4)(16). Overrepresented functional annotations within the data set were provided as GO (Gene Ontology) terms of the category ‘Disease and Function Annotations’ (Ingenuity Pathway Analysis, IPA). Expression data are available at the public repository database GEO, accession number GSE56401[39].

## Supporting Information

S1 Fig CIP2A HOZ mice present similar B cell subpopulations than WT mice. (A) Protein expression of spleen and testis from WT and CIP2AHOZ adult mice, from littermates. 100 μg of total protein lysate were loaded per well. (B) Flow cytometric analysis (Boolean gating) of splenocyte B cell subsets using the following markers: CD5, CD11b, B220, MHC-classII (I/E), IgD, CD95.
(PDF)

S2 Fig CIP2A is involved in mouse sensitivity to infection. (A). Graphic description of the *L*.*m* infection test: primary infection allows to study effects of CIP2A deficiency on early immune responses whereas recall infection leads to analysis of immunological memory. (B) Large abscesses with necrosis developed in liver of WT (1) and CIP2AHOZ (2) mice during primary infection with Listeria monocytogenes. Scale bar equals 1 mm. (C) Bacterial burden (CFU; colony forming units per gram of organ) in spleen from re-infected mice, 4 weeks after a first *L*.*m*.-OVA immunization. *: p = 0.0556, Mann-Whitney t-test.
(PDF)

S3 Fig Analysis of T lymphocytes from WT and CIP2A^HOZ^ mice 5 days after recall infection with high-dose *L.m.*-Ova. (A) Total counts of all splenocytes, CD4+ or CD8+ specific ones, 5 days after recall infection. *: p = 0.0556, Mann-Whitney t-test. (B) Total counts of H2-Kb/SIINFEKL multimer+ cells of CD8+ T cells. p = 0.0571, Mann-Whitney test. (C) TNF-alpha producing cells, gated on CD45^+^ CD3^+^ CD8^+^. (D) CD45^+^ CD3^+^ CD4^+^ gated cells expressing IFN-gamma (left) or TNF-alpha (right). (E) Bar chart numbers indicate percentages of central memory T-cell (CD127^+^ CD62L^high^) and effector memory T-cells (CD127^+^ CD62L^low^) populations on Ova-specific (H-2Kb/SIINFEKL multimer+) CD8^+^ T cells.
(PDF)

S4 Fig CIP2A expression is induced during T cell activation and modulates this activation. (A) CIP2A mRNA expression from WT or ZAP70 negative Jurkat T cells. (B) Representative CFSE dilution by CD4^+^CD62L^+^ T cells isolated from WT or CIP2A^HOZ^ mice stimulated with anti-CD3 and anti-CD28 after 4 days of culture. (C) Quantification of three biological replicates (paired Student t-test). (D) Representative CFSE dilution by human CD4^+^ T cells isolated from umbilical cord blood (pooled from 5–6 individuals) after nucleofection with scramble non-targeting siRNA or CIP2A siRNA. Cells were rested for 48hrs and activated with anti-CD3 and anti-CD28 after 4 days of culture. (E) Quantification of three biological replicates (paired Student t-test).
(PDF)

S1 File Supplemental Experimental Procedures. Detailed protocols are presented as supporting information.
(DOCX)

S1 Table Number of animals used per organ for RT-Q-PCR analysis. (PDF)
