# Supplementary material for: CIP2A Promotes T-Cell Activation and Immune Response to Listeria monocytogenes Infection
Source: PLoS One. 2016 Apr 21;11(4):e0152996. doi: 10.1371/journal.pone.0152996 (PMC4839633; doi:10.1371/journal.pone.0152996)
Supplement: S1 Fig — (A) Protein expression of spleen and testis from WT and CIP2AHOZ adult mice, from littermates. 100 μg of total protein lysate were loaded per well. (B) Flow cytometric analysis (Boolean gating) of splenocyte B cell subsets using the following markers: CD5, CD11b, B220, MHC-classII (I/E), IgD, CD95. (PDF) [file pone.0152996.s001.pdf]

A

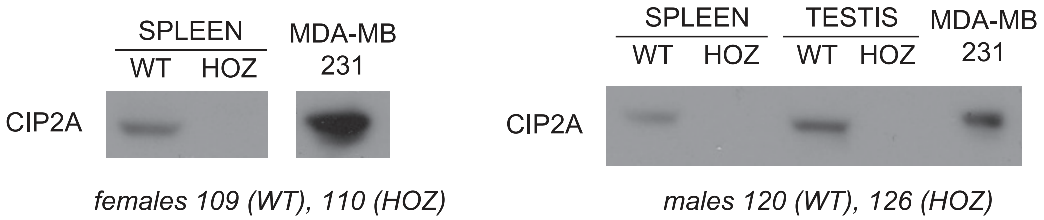

B

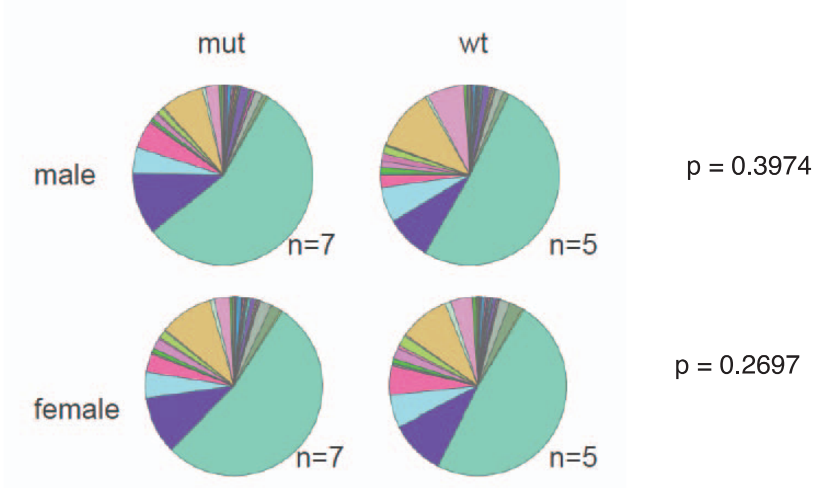

Figure S1: CIP2A^{HOZ} mice present similar B cell subpopulations than WT mice
(A) Protein expression of spleen and testis from WT and CIP2A^{HOZ} adult mice, from littermates. 100 µg of total protein lysate were loaded per well.
(B) Flow cytometric analysis (Boolean gating) of splenocyte B cell subsets using the following markers: CD5, CD11b, B220, MHC-classII (I/E), IgD, CD95.
