# Supplementary material for: CIP2A Promotes T-Cell Activation and Immune Response to Listeria monocytogenes Infection
Source: PLoS One. 2016 Apr 21;11(4):e0152996. doi: 10.1371/journal.pone.0152996 (PMC4839633; doi:10.1371/journal.pone.0152996)
Supplement: S2 Fig — (A). Graphic description of the L.m infection test: primary infection allows to study effects of CIP2A deficiency on early immune responses whereas recall infection leads to analysis of immunological memory. (B) Large abscesses with necrosis developed in liver of WT (1) and CIP2AHOZ (2) mice during primary infection with Listeria monocytogenes. Scale bar equals 1 mm. (C) Bacterial burden (CFU; colony forming units per gram of organ) in spleen from re-infected mice, 4 weeks after a first L.m.-OVA immunization. *: p = 0.0556, Mann-Whitney t-test. (PDF) [file pone.0152996.s002.pdf]

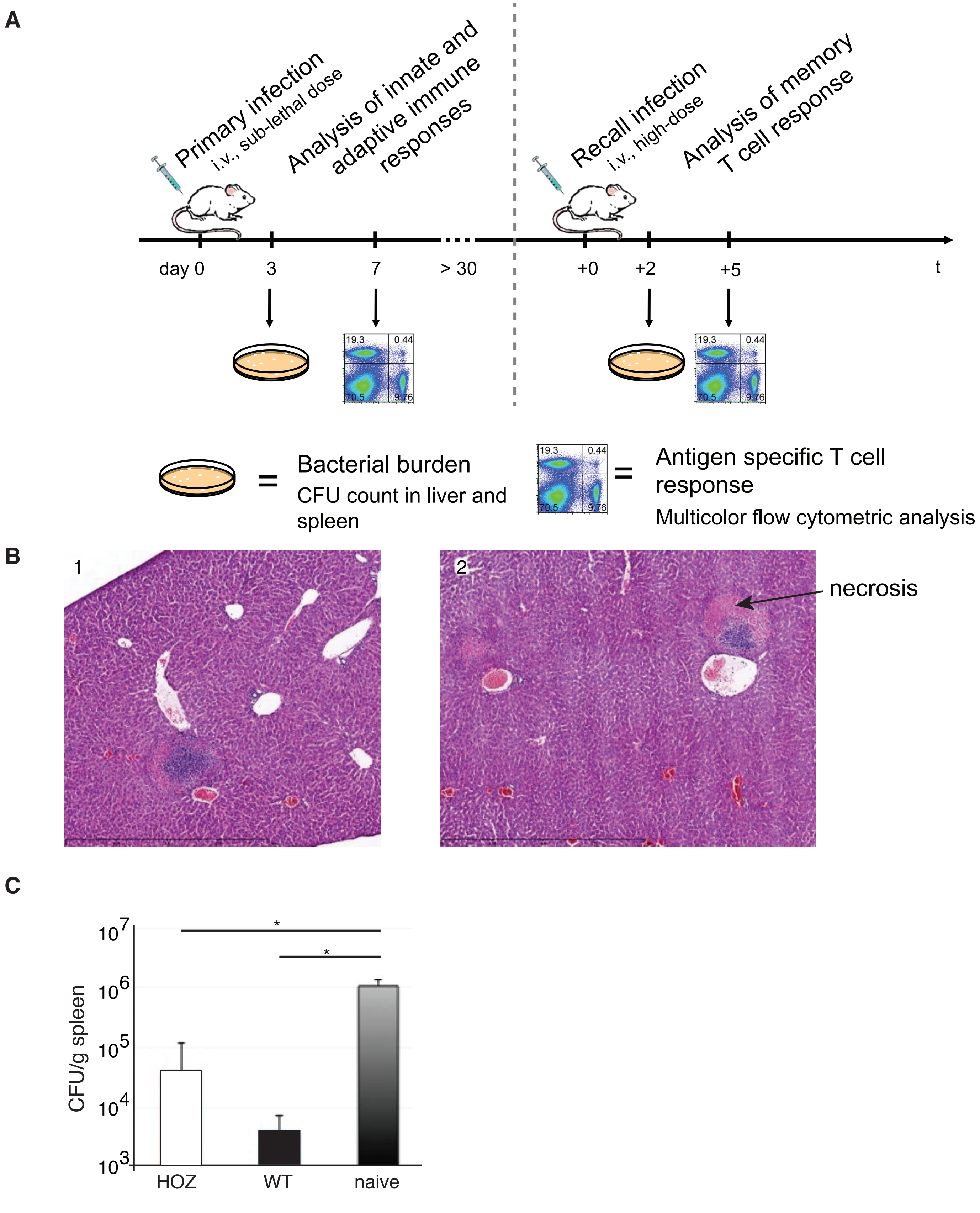

Figure S2: CIP2A is involved in mouse sensitivity to infection

(A) Graphic description of the L.m infection test: primary infection allows to study effects of CIP2A deficiency on early immune responses whereas recall infection leads to analysis of immunological memory.

(B) Large abscesses with necrosis developed in liver of WT (1) and CIP2A^{HOZ} (2) mice during primary infection with Listeria monocytogenes. Scale bar equals 1 mm.

(C) Bacterial burden (CFU; colony forming units per gram of organ) in spleen from re-infected mice, 4 weeks after a first L.m.-OVA immunization. Average from 2 cohorts of mice (3 WT and 3 HOZ, 4 WT and 4 HOZ respectively). *: $p = 0.0556$, Mann-Whitney t-test.
