# Supplementary material for: CIP2A Promotes T-Cell Activation and Immune Response to Listeria monocytogenes Infection
Source: PLoS One. 2016 Apr 21;11(4):e0152996. doi: 10.1371/journal.pone.0152996 (PMC4839633; doi:10.1371/journal.pone.0152996)
Supplement: S3 Fig — (A) Total counts of all splenocytes, CD4+ or CD8+ specific ones, 5 days after recall infection. *: p = 0.0556, Mann-Whitney t-test. (B) Total counts of H2-Kb/SIINFEKL multimer+ cells of CD8+ T cells. p = 0.0571, Mann-Whitney test. (C) TNF-alpha producing cells, gated on CD45+ CD3+ CD8+. (D) CD45+ CD3+ CD4+ gated cells expressing IFN-gamma (left) or TNF-alpha (right). (E) Bar chart numbers indicate percentages of central memory T-cell (CD127+ CD62Lhigh) and effector memory T-cells (CD127+ CD62Llow) populations on Ova-specific (H-2Kb/SIINFEKL multimer+) CD8+ T cells. (PDF) [file pone.0152996.s003.pdf]

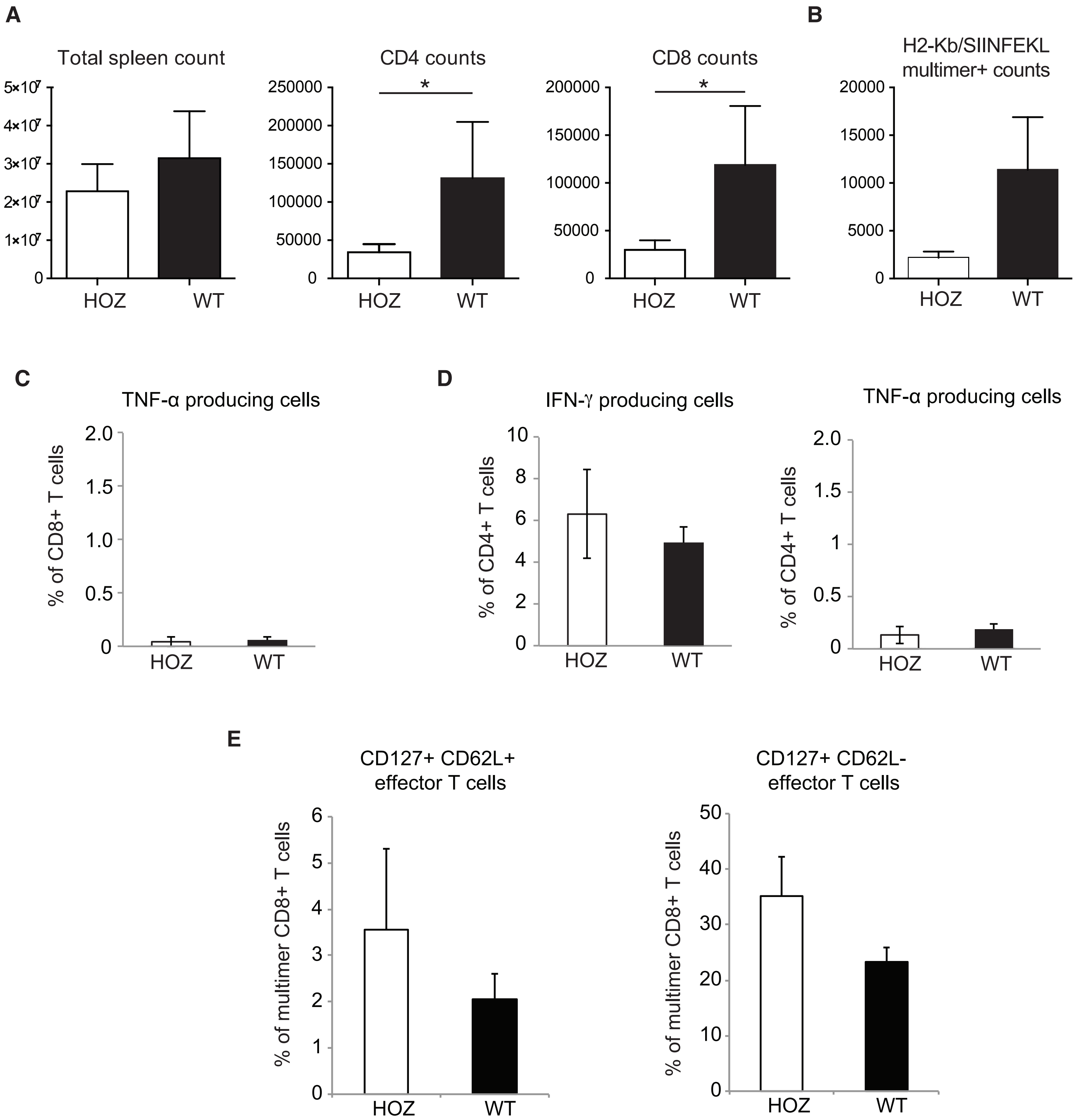

Figure S3: Analysis of T lymphocytes from WT and CIP2A^{HOZ} mice 5 days after recall infection with high-dose L.m.-Ova.

(A) Total counts of all splenocytes, CD4+ or CD8+ specific ones. *: p = 0.0556, Mann-Whitney t-test.

(B) Total counts of H2-Kb/SIINFEKL multimer+ cells of CD8+ T cells. p = 0.0571, Mann-Whitney test.

(C) TNF-alpha producing cells, gated on CD45+ CD3+ CD8+.

(D) CD45+ CD3+ CD4+ gated cells expressing IFN-gamma (left) or TNF-alpha (right).

(E) Bar chart numbers indicate percentages of various effector T-cell population (CD127+ CD62Lhigh and CD127+ CD62Llow) populations on Ova-specific (H-2Kb/SIINFEKL multimer+) CD8+ T cells.
