# Supplementary material for: CIP2A Promotes T-Cell Activation and Immune Response to Listeria monocytogenes Infection
Source: PLoS One. 2016 Apr 21;11(4):e0152996. doi: 10.1371/journal.pone.0152996 (PMC4839633; doi:10.1371/journal.pone.0152996)
Supplement: S4 Fig — (A) CIP2A mRNA expression from WT or ZAP70 negative Jurkat T cells. (B) Representative CFSE dilution by CD4+CD62L+ T cells isolated from WT or CIP2AHOZ mice stimulated with anti-CD3 and anti-CD28 after 4 days of culture. (C) Quantification of three biological replicates (paired Student t-test). (D) Representative CFSE dilution by human CD4+ T cells isolated from umbilical cord blood (pooled from 5–6 individuals) after nucleofection with scramble non-targeting siRNA or CIP2A siRNA. Cells were rested for 48hrs and activated with anti-CD3 and anti-CD28 after 4 days of culture. (E) Quantification of three biological replicates (paired Student t-test). (PDF) [file pone.0152996.s004.pdf]

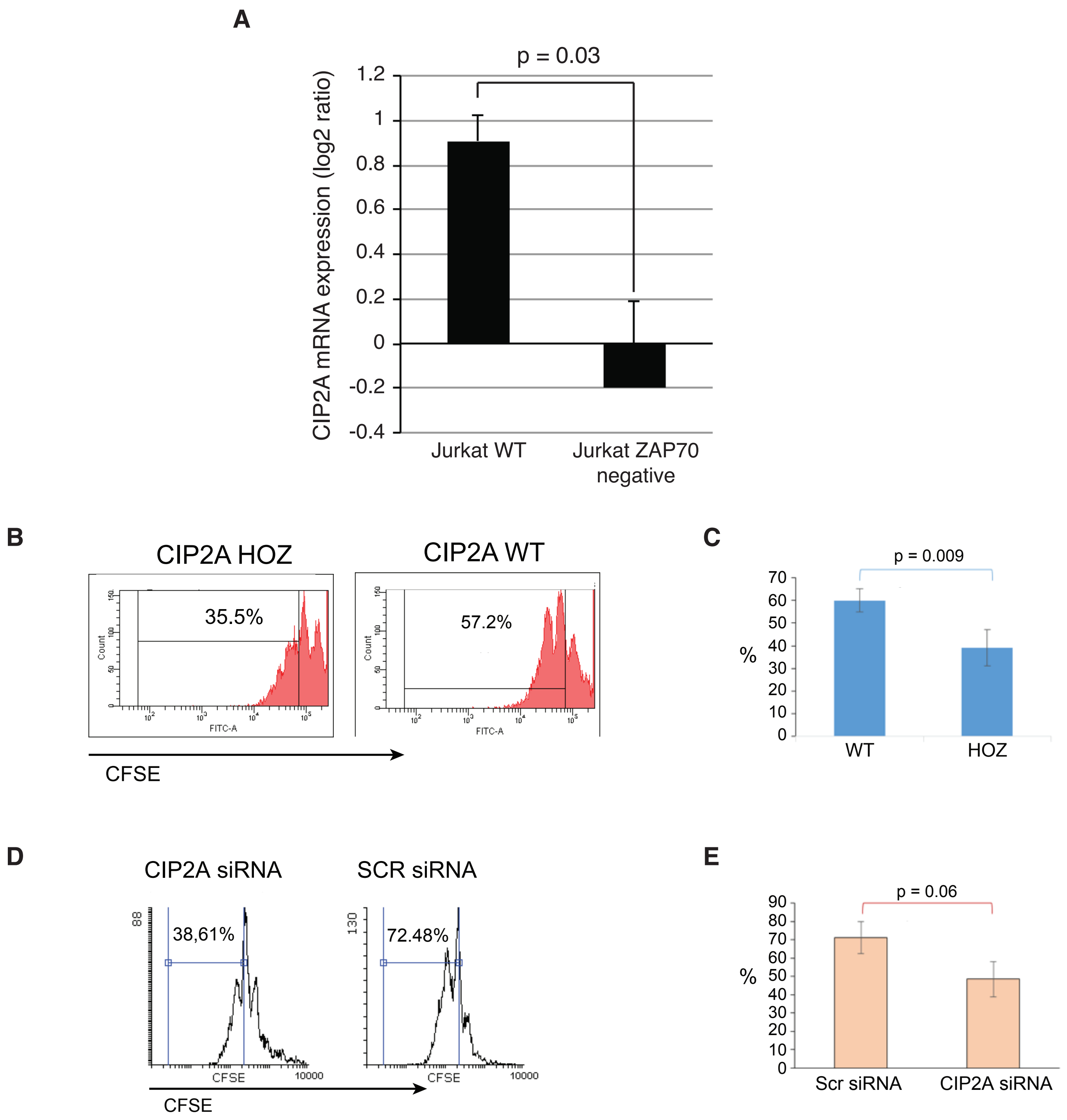

Figure S4: CIP2A expression is induced during T cell activation and modulates this activation

(A) CIP2A mRNA expression from WT or ZAP70 negative Jurkat T cells.

(B) Representative CFSE dilution by $CD4^{+}CD62L^{+}$ T cells isolated from WT or $CIP2A^{HOZ}$ mice stimulated with anti-CD3 and anti-CD28 after 4 days of culture. (C) Quantification of three biological replicates (paired Student t-test).

(D) Representative CFSE dilution by human $CD4^{+}$ T cells isolated from umbilical cord blood (pooled from 5-6 individuals) after nucleofection with scramble nontargeting siRNA or CIP2A siRNA. Cells were rested for 48hrs and activated with anti-CD3 and anti-CD28 after 4 days of culture. (E) Quantification of three biological replicates (paired Student t-test).
