# Supplementary material for: CIP2A Promotes T-Cell Activation and Immune Response to Listeria monocytogenes Infection
Source: PLoS One. 2016 Apr 21;11(4):e0152996. doi: 10.1371/journal.pone.0152996 (PMC4839633; doi:10.1371/journal.pone.0152996)
Supplement: S1 File — Detailed protocols are presented as supporting information. (DOCX) [file pone.0152996.s005.docx]

**Supplementary information** (Come et al; CIP2A promotes T cells activation and facilitates immune response to *Listeria Monocytogenes* infection)

**Supplemental Experimental Procedures**

Immunohistochemistry and tissue samples (detailed)

Formalin-fixed, paraffin embedded sections of mouse organs were cut into 6 mm thin sections, deparaffinized and thereafter rehydrated. Epitope retrieval was then proceeded in 10 mM Tris-EDTA-buffer pH 9,0 during 4 min in microwave oven 4 min 850 W followed by 15 min 100 W. After cooling down for 20 min at room temperature (RT), samples were rinsed properly in water. Concerning the staining procedure, protein of the slides were firstly blocked for 10 min in 3% BSA PBS. After rinsing in Tris- HCl pH 7,4, incubation of primary antibody with CIP2A (1:10000 rabbit polyclonal anti-CIP2A) [[1](#_ENREF_1)], ki-67: (1:5000 mouse monoclonal anti-ki67 (M7249, Dako)) was done for 60 min in 3% BSA/PBS or overnight in immunohistochemical staining. Control slides were incubated with normal non-immunized appropriate animal serum. Following few rinses, secondary antibody (Dako EnVision anti-rat) was done for 30 min. Slides, again rinsed, were then incubated in DAB+ liquid Dako (K3468) for 10 min, and then rinsed in water. Samples were incubated in Mayers HTX for 1 min, rinsed with tap water, and finally dehydrated, cleared and mounted. For peanut agglutinin (PNA) staining, the following protocol was used, all steps at RT and based on a basic buffer containing 0,05% Tween in PBS (PBST). Slides were blocked in 5% BSA PBST for 30 minutes, followed by incubation with biotinylated PNA (L6135, Sigma-Aldrich) at 4°C, overnight, in 5% BSA PBST. 3 washes of 3 minutes were then applied, followed by an immersion in 3% H_2_O_2_ for 10 minutes. After new washing, slides were incubated in HRP conjugated streptavidin from the LSAB+ skit (K0690, Dako) for 30 minutes. Slides, again rinsed, were then incubated in DAB+ liquid Dako (K3468) for 10 min, and then rinsed in water. Samples were incubated in Mayers HTX for 1 min, rinsed with tap water, and finally dehydrated, cleared and mounted.

Immunology screen, blood samples

Blood samples were collected from isoflurane-anesthetized mice (14 weeks old; 10 WT, 10 CIP2A^HOZ^, for each gender) by puncturing the retro-bulbar sinus with non-heparinized glass capillaries (1.0 mm in diameter; Neolab; Munich, Germany). Blood was collected in a heparinized tube (Li-heparin, KABE, Art.No. 078028; Nümbrecht, Germany). Each tube was immediately inverted five times to achieve a homogeneous distribution of the anticoagulant and then left in a rack at RT for one to two hours. Afterwards, cells and plasma were separated by a centrifugation step (10 min, 5000g; 8°C, Biofuge fresco, Heraeus; Hanau, Germany). Plasma was then collected while the cell pellet was used for FACS-analysis. From this pellet, frequencies of the main circulating peripheral blood leukocytes (PBLs) were measured by flow cytometry. PBLs were isolated from the cell pellet of 500 µl whole blood samples after centrifugation. The cell pellet is dissolved in 600 µl NH4Cl-based, Tris-buffered erythrocyte lysis solution, and 150 µl transferred into 96-well micro titer plates. After subsequent washing steps with FACS staining buffer (PBS, 0.5%BSA, 0.02%sodium azide, pH 7.45), PBLs were incubated for 20 min with Fc block (clone 2.4G2, PharMingen, San Diego, USA). Cells were then stained with fluorescence-conjugated monoclonal antibodies (PharMingen). After the antibody incubation, propidium iodide was added for the identification of dying/dead cells [[2](#_ENREF_2)] which might bind antibodies unspecifically, and/or loose specific antigens upon apoptosis [[3](#_ENREF_3)]. Samples were acquired from 96 well plates and measured with a three laser 10-color flow cytometer (LSRII, Becton Dickinson, USA; Gallios, Beckman Coulter, USA). A total number of 10 000 - 30 000 living CD45+ per sample is reached. For analysis, intact cells are first identified by their FSC/SSC profile. These cells were gated on the basis of their propidium iodide/PE signal (compensated parameters), allowing the dead cells to be gated out. Living cells were then gated using their SSC/CD45 signal, gating out remaining erythrocytes, thrombocytes and debris [[4](#_ENREF_4)]. CD45+ cells are subsequently analyzed by software based analysis (Flowjo, TreeStar Inc, USA; SPICE [[5](#_ENREF_5)]). In former experiments, FMO (Fluorescence minus one) controls from wild-type mice have been used to define ’positive’ and ’negative’ regions [[6](#_ENREF_6)]. The following stainings were performed to identify the different leukocytes populations: staining 1: T cells (CD3+), CD4+ T cells, CD8+ T cells, γδT cells (CD3+ gdTCR+), T reg cells (CD4+CD25+); staining 2: NK cells (NKp46 and/or NK1.1+ CD5-), B cells (CD19 and/or B220+), B1 B cells (CD19+CD5+), granulocytes (CD11b+Gr1+), NK T cells (NKp46 and/or NK1.1 CD5+), monocytes (non NK cells, non granulocytes) and CD11b+. Based on the previous stainings, few subpopulations were analyzed: staining 1: T cells: CD62L+, CD44+ expressing cells; Staining 2: B cells: IgD+, MHC-class II+; NK cells: CD11b+. Finally, a last set of further subpopulations were identified and analyzed by Boolean and bi-variate gating using the following markers: staining 1: CD25, CD62L, CD44, Ly6C; staining 2: CD11b, MHC class I.

## Listeria monocytogenes infection (detailed)

Infection experiments were performed with recombinant *Listeria monocytogenes* expressing ovalbumin (L.m.-Ova, [[7](#_ENREF_7)]) or the parental wild type *Listeria* *monocytogenes* strain 10403s (L.m.-wt). Brain Heart Infusion (BHI) medium was inoculated with listeria stock solution and incubated at 37°C until an OD600 of 0.05 - 0.1. After dilution with PBS to an appropriate concentration the infection of mice was performed with the indicated dose by intravenous (i.v.) injection into the lateral tail vein. S2A Figure represents a graphic description of the different tests performed, which are detailed here after.

** Susceptibility to L.m. infection*

The innate immune response restricts substantially the bacterial growth after primary infection and is essential for the survival of infected mice [[8](#_ENREF_8)]. Mice were infected with 50 000 L.m.-wt and in order to assess the competence of their innate immunity, the numbers of viable bacteria in spleen and liver of infected mice were measured 3 days after infection.

* *Re-call infection related assays*

Infection with a sublethal dose of L.m. induces memory T cells that mediate long-lasting specific protective immunity to subsequent re-infection [[9-11](#_ENREF_9)]. Mice were immunized with a low-dose of 5,000 L.m.-Ova; 4 weeks after immunization mice were challenged with a lethal dose of 500,000 L.m-Ova.

- *Protection assay*

Splenic growth of L.m. was quantified on day 2 after re-infection by measuring bacterial burden.

- *Memory T cell response*

On day 5 after recall infection we performed a multimer (H-2Kb/SIINFEKL) staining, which identifies L.m. ova-specific CD8+ T cells; and also analyzed cytokine production by intracellular cytokine staining upon in vitro restimulation with the immunodominant epitopes for CD8+ or CD4+ T cells (ova-peptides SIINFEKL or ova190-201, respectively).

Short-living effector T cells express low amounts of the IL-7 receptor alpha chain (CD127) and L-selectin (CD62L) on their surface. The long-living memory T cell pool is composed of at least two distinct subtypes: central memory (T_cm_) and effector memory (T_em_) T cells. Memory T cells upregulate CD127; CD62L expression is used to discriminate TCM (CD62L^high^) from TEM (CD62L^low^) [[12](#_ENREF_12)].

- *Antigen specific T cell response*

Specific T cells are needed for final elimination of the pathogen [[13](#_ENREF_13)]. In response to L.m. infection, naïve CD8+ T cells proliferate and acquire effector functions. Studies of Listeria infection in mice deficient for interferon (IFN)-γ [[14](#_ENREF_14)], IFN- γ receptor [[15](#_ENREF_15)] or tumor necrosis factor (TNF) receptor 1 [[16](#_ENREF_16)] have shown that two cytokines, IFN-γ and TNF-α, are crucial for survival. On day 5 after recall-infection, we analyzed cytokine production by CD8+ T cells upon *in vitro* antigen-specific peptide restimulation.

Our listeria challenge concept was designed to minimize the suffering of animals:

- For the evaluation of the primary resistance to L.m infection, all animals were euthanized on day 3 after infection, before any signs of disease (such as tousled fur) could be detected. The day 3 was selected as a time point as the biological response can already be analyzed and mice do not present any significant stress, usually appearing at days 4-6 post-infection. During this experiment, health status of the mice was checked twice a day.

- Regarding the analysis of the T cell response, low doses were used. In immuno-competent mice, on day 7 (day of the euthanasia and analysis) L.m. are sufficiently cleared, and the mice do not show any signs of disease. We performed daily health check during this experiment.

- For the memory T cell response, we immunized mice with a low dose of L.m. as well. In previously immunized mice, the second infection, with a dose that would have been lethal for non-immunized animals, is rapidly cleared by memory T cells. Mice show no signs of disease, thus the suffering of animals is low. We performed a daily health check of the mice.

All animals were in good health conditions throughout all experiments, no unexpected deaths occurred. Therefore, no anesthesia, analgesics or anesthetics were used.

We euthanized mice on the day of analysis by cervical dislocation.

**References**

1. Hoo LS, Zhang JY, Chan EK (2002) Cloning and characterization of a novel 90 kDa ‘companion’ auto-antigen of p62 overexpressed in cancer. Oncogene 21: 5006-5015.

2. Zamai LL, Falcieri EE, Marhefka GG, Vitale MM (1996) Supravital exposure to propidium iodide identifies apoptotic cells in the absence of nucleosomal DNA fragmentation. Cytometry Part A 23: 303-311.

3. Diaz D, Prieto A, Barcenilla H, Monserrat J, Prieto P, et al. (2004) Loss of lineage antigens is a common feature of apoptotic lymphocytes. Journal of leukocyte biology 76: 609-615.

4. Weaver JL, Broud DD, McKinnon K, Germolec DR (2001) Serial phenotypic analysis of mouse peripheral blood leukocytes. Toxicology Mechanisms and Methods 12: 95-118.

5. Roederer M, Nozzi JL, Nason MC (2011) SPICE: Exploration and analysis of post-cytometric complex multivariate datasets. Cytometry Part A 79A: 167-174.

6. Baumgarth N, Roederer M (2000) A practical approach to multicolor flow cytometry for immunophenotyping. Journal of immunological methods 243: 77-97.

7. Pope C, Kim SK, Marzo A, Masopust D, Williams K, et al. (2001) Organ-specific regulation of the CD8 T cell response to Listeria monocytogenes infection. J Immunol 166: 3402-3409.

8. Pamer EG (2004) Immune responses to Listeria monocytogenes. Nat Rev Immunol 4: 812-823.

9. Harty JT, Bevan MJ (1992) CD8+ T cells specific for a single nonamer epitope of Listeria monocytogenes are protective in vivo. J Exp Med 175: 1531-1538.

10. Kägi D, Ledermann B, Bürki K, Hengartner H, Zinkernagel RM (1994) CD8+ T cell-mediated protection against an intracellular bacterium by perforin-dependent cytotoxicity. European Journal of Immunology 24: 3068-3072.

11. M E Mielke SEHH (1988) T-cell subsets in delayed-type hypersensitivity, protection, and granuloma formation in primary and secondary Listeria infection in mice: superior role of Lyt-2+ cells in acquired immunity. Infection and Immunity 56: 1920.

12. Huster KM, Busch V, Schiemann M, Linkemann K, Kerksiek KM, et al. (2004) Selective expression of IL-7 receptor on memory T cells identifies early CD40L-dependent generation of distinct CD8+ memory T cell subsets. Proceedings of the National Academy of Sciences of the United States of America 101: 5610-5615.

13. Bancroft GJ, Schreiber RD, Bosma GC, Bosma MJ, Unanue ER (1987) A T cell-independent mechanism of macrophage activation by interferon-gamma. J Immunol 139: 1104-1107.

14. Dalton DK, Pitts-Meek S, Keshav S, Figari IS, Bradley A, et al. (1993) Multiple defects of immune cell function in mice with disrupted interferon-gamma genes. Science 259: 1739-1742.

15. Huang S, Hendriks W, Althage A, Hemmi S, Bluethmann H, et al. (1993) Immune response in mice that lack the interferon-gamma receptor. Science 259: 1742-1745.

16. Rothe J, Lesslauer W, Lotscher H, Lang Y, Koebel P, et al. (1993) Mice lacking the tumour necrosis factor receptor 1 are resistant to TNF-mediated toxicity but highly susceptible to infection by Listeria monocytogenes. Nature 364: 798-802.
