# Supplementary material for: CIP2A Promotes T-Cell Activation and Immune Response to Listeria monocytogenes Infection
Source: PLoS One. 2016 Apr 21;11(4):e0152996. doi: 10.1371/journal.pone.0152996 (PMC4839633; doi:10.1371/journal.pone.0152996)
Supplement: S1 Table — (PDF) [file pone.0152996.s006.pdf]

Table S1: Number of animals used per organ for RT-Q-PCR analysis

organ	WT	$CIP2A^{HOZ}$
Bone marrow	5	8
Lymph Node	8	5
Spleen	11	19
Thymus	3	3
Testis	2	4
